# Tillage, green manuring and crop residue management impacts on crop productivity, potassium use efficiency and potassium fractions under rice-wheat system

**DOI:** 10.1016/j.heliyon.2023.e17828

**Published:** 2023-06-29

**Authors:** Sandeep Sharma, Pritpal Singh, Hayssam M. Ali, Manzer Hussain Siddiqui, Javed Iqbal

**Affiliations:** a Department of Soil Science, Punjab Agricultural University, Ludhiana 141004, India; b Department of Botany and Microbiology, College of Science, King Saud University, Riyadh 11451, Saudi Arabia; c Department of Agronomy and Horticulture, University of Nebraska–Lincoln, USA

**Keywords:** Rice residue, Green manure, Potassium fractions, Potassium use efficiencies and crop yield

## Abstract

The conventional crop production practices including intensive tillage and open field crop residue burning in world’ largest rice-wheat system (RWS) are adversely affecting crop productivity besides deteriorating natural resources and ecosystems’ sustainability. In order to improve system productivity, potassium (K) use efficiency and apparent K balance, adoption of conservation tillage in a RWS with residue management is considered highly effective. We therefore, studied the effect of wheat straw retention and green manure (GM) in rice (main plot treatment), and tillage and rice residue management in subsequent wheat (sub-plot treatments) on crop productivity, K use efficiency and its transformation amongst different fractions of variable solubility. These results revealed that rice straw retention along with GM significantly (*p* < 0.05) increased the rice yields by ∼5.3–6.7% and wheat yields by ∼10.2–16.9%, compared to the conventional tillage (CT) without GM. Green manuring during the intervening period (CTR_W0_+GM) significantly increased the rice grain K uptake by ∼36.2% than in plots with no-GM (CTR_W0_). However, it increased by ∼29.8% under CTR_W25_+GM, compared with CTR_W25_-GM treatment. As compared with CTR_W0_, CTR_W0_+GM significantly increased the reciprocal internal use efficiency of K of rice by 3.8 kg Mg^−1^ grain yield (∼29.5%). However, CTR_W25_+GM increased the RIUE_K_ of rice by 3.3 kg Mg^−1^ grain yield (∼22.4%), compared with no-GM (CTR_W25_). Although, apparent K balance was net negative for CTR_W25_, ZTW_R100_ treatments, yet there was decreased K mining of 56–262 kg K ha^−1^ (∼11.9–61.2%) for CTR_W25_ and ZTW_R100_ over CTR_W0_ and ZTW_R0_. The increased crop yield, K uptake and K use efficiency were significantly related to K enrichment in water soluble K, exchangeable K, non-exchangeable-K, hydrochloric acid extractable-K, lattice-K and total K fractions by ∼1.3, 3.4, 18.6, 11.0 and 34.1%, respectively due to residue retention, compared with no residue. Therefore, conventional tillage with puddled transplanted rice (CTR) with wheat residue and green manure during intervening period (CTR_W25_+GM), and zero tillage wheat with rice residue retention (ZTW_R100_) were emerged as highly valuable technological options for mitigating soil degradation effects under intensive RWS for food grains in north-western India.

## 1 Introduction

Rice-wheat system (RWS) is an important cropping system for the food, nutritional and income security of millions of people due to wide spread adoption and coverage of ∼10.5 million ha (Mha) in the Indo-Gangetic Plains (IGPs) of India [1,2]. The RWS has become highly tillage intensive [3,4] with significantly higher energy-, water- and carbon (C) footprints [5,6], besides deterioration in soil health [7], decreased C sustainability [8], and occurrence of multiple macro-and micro-nutrients’ deficiencies due to imbalanced use of fertilizers [9]. Nonetheless, the foremost important unsustainability has been the open field burning of crop residues, particularly of rice before establishment causing air pollution due to subsequent emission of greenhouse gases (GHGs) [5,10,11]. Recent estimates revealed that annually ∼25 million tons of rice residues are burnt in the IGPs, which has significant environmental implications besides deterioration of soil health due to loss of organic C [12,13].

The conventional tillage (CT) practiced before wheat establishment for seed bed preparation is considered highly intensive [8], and often lead to deterioration of soil health due to decreased organic C pool causing serious environment pollution [14,15]. Zero tillage (ZT) with residue retention on soil surface significantly improve overall soil physical, chemical and biological health through replenishing soil organic matter [[14], [15], [16], [17]], which lead to increase in availability of nutrients to support sustainable RWS [18]. Therefore, *in-situ* crop residue management has become necessity to arrest nutrient loss, increased C accrual, mitigation of GHGs emissions for assured sustainability of a cropping system. Although, soils of IGPs regions of India are rich in K, yet native soil K is soils under RWS has been declining due to intensive cultivation [19]. *In-situ* crop residue management as a part of conservation agriculture (CA) has been an important K source [20]. Potassium is critically important for crops [21] therefore, insufficient K supply can lead to a significant loss in grain yield. The above-ground crop residues biomass has ability to return large portion of nutrients [22]. Rice assimilated more K than wheat and rice straw has higher K concentration than grains [23], which indicates that rice needs more K to produce higher yields. Potassium is not a structural component of the plant and therefore, easily released from plant tissue upon decomposition and become readily available in the soil [24]. Nonetheless, K being an essential nutrient is required for numerous plants metabolic activities, cellular osmoregulation processes, resistance to biotic and abiotic stresses and enhancing nutrients use efficiency [5,19,[25], [26], [27]]. About 75–80% of the total K removal by plant from soils is retained in the cereal straw [28]. A 30-years field trial conducted by Liao et al. [29] indicated that straw management could increase exchangeable and non-exchangeable K fractions and organic C pool by ∼26.4, 1.8 and 21.0%, respectively than without straw in rice soils. Yadvinder-Singh et al. [30] reported that release of K from rice straw increases K availability in soils by ∼32% in straw amended treatments than untreated control within just 10 days after incorporation. In another study, ∼85–88% of K from incorporated rice residue was released by maximum tillering of succeeding wheat crop in RWS, whereas almost entire K (>97% of total K in rice straw) was released during the wheat season in a RWS on course textured Inceptisol of Ludhiana, India [31], suggesting that K release from rice residue incorporated during wheat season [19].

In soil, total K pool mainly exists in four different fractions viz. water soluble K (WS–K), exchangeable K (Ex-K), non-exchangeable K (Non Ex-K) and the lattice-K, which stay in dynamic equilibrium and controls the K availability to plants [15,32]. A change in equilibrium among different forms depends on nutrient management practices, and their likely impact on pool size of each K fraction [33]. Increase in WS-K and Ex-K fractions while a decrease in NonEx-K fraction and total K pool in soils under RWS in alluvial soils of IGPs for 15-years has been reported earlier [34]. The crop residue removal without adequately replenishing K with mineral fertilizers has been reported to exacerbate K deficiency in soils, especially those under RWS [35]. Therefore, the management of crop residues especially of rice residue highly rich in K for improving soil health and crop productivity is important. The long-term studies conducted recently suggest that inadequate supply of K has been one of the major reasons which limit sustainable production in the entire Indian IGPs [36]. In India, generally 40–60 kg K_2_O ha^−1^ is recommended to different cereal crops, which is far less than the amount of K removed by crops particularly when crop residues are not returned to the soil [30,37]. Therefore, in most of the cereal based cropping systems, a negative K balance exists [38,39], particularly in intensively cultivated regions [19]. Earlier studies showed considerable changes in WS-K, Ex-K and NonEx-K forms due to manure application and/or residue incorporation into the soil [40,41], and improved the K use efficiency [19,42]. The incorporation of GM is eco-friendly, nutrient-rich, and easily biodegradable releases potassium [43,44] and improve root growth for better K uptake by the plants [45]. Likewise, GM enhances the microbial action for rapid decomposition process and improves the availability of K to plants [46]. Till date, the information on effect of combined application of tillage, green manure and residue retention on crop yields and K use efficiency due to change in different K forms in soils is limited [47]. We hypothesized that tillage, GM and residue retention would leads to dynamically change the dynamics between different K forms to influence the K use efficiency. The present was conducted to study the change in different K forms in a sandy loam soils and its influence on K use efficiency in highly intensive RWS in north-western India.

## 2 Materials and methods

### 2.1 Brief description of experimental site

A 7-years field experiment on irrigated RWS was established in 2011 starting with rice on a sandy loam soil (135 g clay, 160 g silt and 705 g sand kg^−1^); *Typic Ustochrept* as per USDA classification at the research farm of Punjab Agricultural University (P.A.U.), Ludhiana, Punjab located in India at an elevation of ∼247 m above mean sea level (30°54' latitude and 75°40' longitude). The surface (0–15 cm) soil layer at the beginning of experiment has low concentration of soluble salt (electrical conductivity; E.C._1:2_ = 0.34 dS m^−1^) with pH_1:2_ = 7.81 and low organic C (3.51 g kg^−1^), 11.3 mg NaHCO_3_-extractable P kg^−1^ and 46.3 mg NH_4_OAc-extractable K kg^−1^. The average annual rainfall in the study region is ∼733 mm; of which ∼78–80% is received between July–September, while the remaining in the winter season. Mean monthly lowest is 13.7 °C in January, while the maximum is 42.2 °C in May.

### 2.2 Experimental design and treatments

The experiment was commenced in a split plot design with three replications. Therefore, thus there were two factors (wheat straw and GM (*Sesbania aculeate*) in rice and tillage and residue management in subsequent wheat crop) (Table 1). Full details of the experimental site, treatments combination and crop management practices are provided in Sakia et al. [48].

**Table 1:** Brief description of treatments and crop establishment methods.

Treatment description	Acronyms	Crop establishment method
Rice (main plot treatments)		
Conventional tillage with puddled transplanted rice (CTR) + wheat residue removal	CTR_W0_	Residue of wheat crop was removed. Field preparation (pre wet-tillage) includes 2 cultivators + 2 disc harrow operations followed by land levelling with wooden planker. Wet tillage (puddling) with tractor mounted puddler was performed twice in 6–8 cm of standing water, followed by planking. Rice seedlings were manually transplanted at 15 × 20 cm spacing (plant density = 33 hills m^−2^).
CTR + wheat residue retention	CTR_W25_	Anchored (∼10–12 cm high) wheat straw (25%) of preceding crop was retained in the field. All the tillage and rice crop establishment operations were same as for PTR_W0_
CTR + green manuring	CTR_W0_+GM	Residue of wheat crop was removed; *Sesbania aculeate* (*dhaincha*) green manure was after wheat with zero till (ZT). Green manuring was done after 6–8 weeks of sowing, chopped with two passes of disc harrows and incorporated into soil with two passes of cultivators. All the tillage and rice crop establishment operations were same as for CTR_W0_
CTR + wheat residue retention + green manuring	CTR_W25_+GM	Anchored (∼10–12 cm high) wheat straw (25%) of preceding crop was retained in the field. All the tillage and *Sesbania aculeate* and rice crop establishment operations were same as for CTR_W0_+GM
Wheat (sub-plot treatments)		
CT wheat + rice residue removal	CTW_R0_	All the residue of previous rice crop was removed. Tillage operations included two passes of disc harrows and two passes of tyne plough followed by land levelling with wooden planker. After pre-sowing irrigation, seed bed was prepared by two passes of tyne plough followed by planking. Wheat crop was sown using seed-cum-fertilizer drill in rows 20 cm apart.
ZT wheat + rice residue removal	ZTW_R0_	All the residue of previous rice crop was removed. Wheat was directly seeded in the no-till plots in rows 20 cm apart using ZT seed-cum-fertilizer drill.
ZT wheat + rice residue retention	ZTW_R100_	All residue of previous rice crop was retained. Wheat was directly seeded in rows 20 cm apart into rice residues using Turbo Happy Seeder.

### 2.3 Collection, preparation and analysis of soil

After completion of 7 cycles of RWS, soil samples were collected from surface soil depth (0–15 cm) with the help of a post-hole auger (inner diameter = 7.1 cm) from three replicates of each treatment. The samples were analysed for total K and its different forms viz. WS-K, Ex-K, Non-Ex-K, 1 N hydrochloric acid extractable-K (HCl–K) and lattice-K following standard procedure, for which brief information could be found in our previously published research [19].

### 2.4 Plant analysis

Wheat grain and straw samples were collected at the time of harvest and oven-dried at 65 °C for 48 h. Total K content was determined by digested in a triple-acid (HNO_3_: H_2_SO_4_: HClO_4_:10:3:1), diluted adequately and analysed for total K using flame photometer [49].

### 2.5 Potassium use efficiency

Two different measures of K use efficiency viz. partial factor productivity of K (PFP_K_) and reciprocal internal use efficiency of K (RIUE_K_) were estimated using following equations (Eq. 1-2).

(1) $${PFP}_{K}\left(kg\;grain\;{kg}^{-1}\;K\;added\right)=\frac{Grain\;yield\;\left({kg\;ha}^{-1}\right)}{Total\;K\;added\;\left({kg\;ha}^{-1}\right)}$$

(2) $${RIUE}_{K}\left(kg\;{Mg}^{-1}grain\;yield\right)=\frac{K\;uptake\;\left({kg\;ha}^{-1}\right)}{Grain\;yield\;\left({Mg\;ha}^{-1}\right)}$$

### 2.6 Statistical analysis

The data were analysed using analysis of variance (ANOVA) with wheat straw and GM in rice and tillage and rice management in subsequent wheat as sub-plot in the split plot design at *p* < 0.05 statistically significant using IBM SPSS for Windows 21.0. The correlations matrix between the variables was assessed by determining *Pearson’s* correlation coefficients (r) and probabilities. The partial K budget for a RWS was estimated from the difference in cumulative (2011–18) total K input and the K uptake by crops.

## 3 Results

Rice and wheat establishment treatments and study years interaction effect on crop grain yield and K uptake were significant (*p* < 0.05), therefore, only main effects of the treatments are discussed in the following sub-sections.

### 3.1 Rice grain and straw yield, and harvest index

Rice grain yield was significantly (*p* < 0.05) higher in CTR_W25_+GM treatment, compared with the other treatments without GM (CTR_W0_/CTR_W25_) (Table 2). Green manuring during the intervening period (CTR_W0_+GM) significantly increased the rice grain yield by ∼8.4%, compared with no-GM treatment (CTR_W0_). Similarly, wheat residue retention with GM treatment (CTR_W25_+GM) significantly increased the rice grain yield by ∼6.7% over CTR_W25_. Wheat residue incorporation with or without GM (CTR_W0_ and CTR_W0_+GM) during intervening period did not significantly increase the rice grain yield over their respective treatments involving residue removal (CTR_W25_ and CTR_W25_+GM). Zero tillage in wheat with rice residue retention (ZTW_R100_) resulted in a significant increase in rice grain yield by ∼5.3%, compared with zero tillage with complete residue removal (ZTW_R0_). Rice grain yield in CTW_R0_ and ZTW_R0_ treatments was statistically at par. Straw yield varied between 6.60 ± 0.39 and 8.00 ± 0.29 Mg ha^−1^ among different treatments; and was significantly higher in CTR_W25_+GM treatment than no-GM (CTR_WR0_). Green manuring during the intervening period in CTR_W0_+GM and CTR_W25_+GM treatments significantly increased the rice straw yield by ∼14.8 and 13.8% respectively over CTR_W0_ and CTR_W25_ treatments. The ZTW_R100_ treatment significantly increased the rice straw yield by ∼8.8% than with ZTW_R0_. Harvest index of rice was significantly decreased with CTR_W25_+GM treatment, compared with CTR_W0_.

**Table 2:** Effect of tillage, crop residue management and green manure on grain yield, straw yield, and harvest index (HI), K uptake by grains and straw and K use efficiency indices viz. partial factor productivity (PFP) and reciprocal internal use efficiency (RIUE_K_) for rice in north-western India.

Treatments^a^	Rice grain yield (Mg ha^−1^)	Rice straw yield (Mg ha^−1^)	Harvest index (HI)	Grain K uptake (kg ha^−1^)	Straw K uptake (kg ha^−1^)	Total K uptake (kg ha^−1^)	PFP	RIUE_K_ (kg Mg^−1^ grain yield)
Rice establishment								
CTR_W0_	6.20a ± 0.18^b^	6.60a ± 0.39	0.49a ± 0.01	23.5a ± 2.3	57.7a ± 7.4	81.3a ± 9.6	248b ± 16	12.9a ± 0.7
CTR_W25_	6.42a ± 0.17	7.03b ± 0.33	0.48a ± 0.01	27.0b ± 2.5	68.7b ± 6.5	95.7b ± 8.7	128a ± 9	14.7b ± 0.9
CTR_W0_+GM	6.72b ± 0.17	7.58c ± 0.40	0.47a ± 0.01	32.0c ± 2.7	81.4c ± 7.7	113.4c ± 10.2	269b ± 24	16.7c ± 1.1
CTR_W25_+GM	6.85b ± 0.13	8.00c ± 0.29	0.46a ± 0.01	34.7d ± 2.3	89.6d ± 6.7	124.3d ± 8.7	134a ± 12	18.0d ± 1.2
Wheat establishment								
CTW_R0_	6.53b ± 0.17	7.19b ± 0.40	0.48a ± 0.01	29.1b ± 2.5	72.4a ± 8.2	101.5b ± 10.5	194a ± 12	15.3a ± 1.2
ZTW_R0_	6.39a ± 0.19	7.04a ± 0.40	0.48a ± 0.01	26.9a ± 2.7	68.7a ± 7.6	95.6a ± 10.1	193a ± 13	14.7a ± 0.9
ZTW_R100_	6.73b ± 0.12	7.66c ± 0.26	0.48a ± 0.01	31.8c ± 2.1	82.0c ± 5.4	113.9c ± 7.3	198a ± 6	16.7b ± 1.2

Mean values for different treatments for each crop establishment method followed by different letters are significantly different at *p* < 0.05 by least significant difference (LSD) test.

a Refer Table 1 for treatment details.

b Values indicate standard error from mean.

### 3.2 Grain, straw and total K uptake by rice

**Fig. 1:**
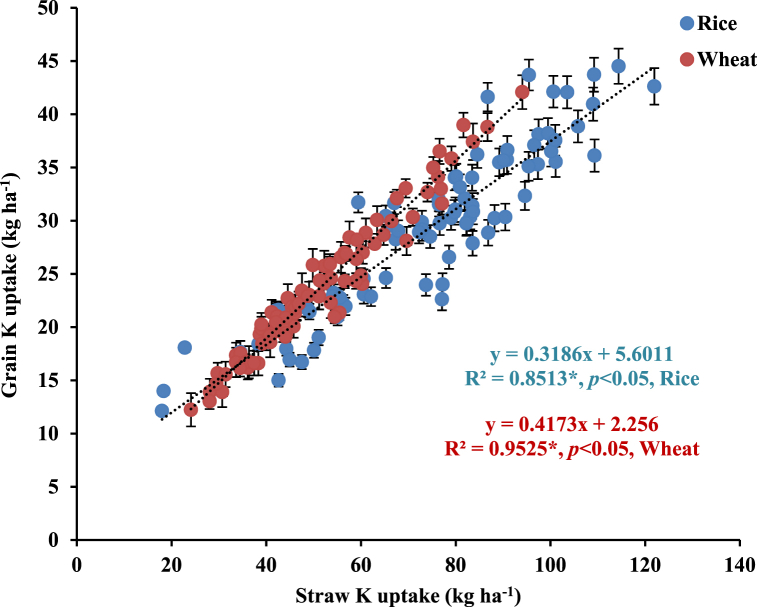
Relationship between grain and straw K uptake by rice and wheat in north-western India. *Error bars indicate standard error from mean*. Data pooled for 7- years (2011/12–2017/18).

The CTR_W25_ treatment significantly increased the rice grain uptake by ∼14.9%, compared with CTR_W0_ (Table 2). Green manuring during the intervening period (CTR_W0_+GM) significantly increased the rice grain K uptake by ∼36.2% than without GM (CTR_W0_). Grain K uptake by rice increased by ∼29.8% wheat with residue retention and GM during the intervening period (CTR_W25_+GM), compared with the treatments where no-GM was done (CTR_W25_-GM). Unsurprisingly, the comparison of CTW_R0_ and ZTW_R0_ revealed ∼8.2% higher rice grain K uptake under CTW_R0_. Rice straw K uptake varied between 57.7 ± 7.4 and 89.6 ± 6.7 kg ha^−1^; and was significantly increased with wheat residue retention and GM during the intervening period. Alone wheat residue retention (CTR_W25_) significantly increased the rice straw K uptake by 11.1 kg ha^−1^ (∼19.1%) than with CTR_W0_. However, wheat residue retention and GM (CTR_W25_+GM) increased the rice straw K uptake by ∼10.1%, compared with CTR_W0_+GM. There was non-significant difference between straw K uptake among ZTW_R0_ and CTW_R0_ treatments. But, wheat residue retention under ZTW_R100_ treatment increased rice straw K uptake by 13.3 kg ha^−1^ (∼19.4%) than with residue removal (ZTW_R0_). These results revealed linear significant increase in grain K uptake with straw K uptake by rice (Fig. 1). The relationship between grain and straw K uptake could best be described by Eqs. (3) and (4).

(3) Rice grain K uptake (kg ha^−1^) = 0.3186 (rice straw K uptake; kg ha^−1^) + 5.6011, R^2^ = 0.8513*, *p* < 0.05

(4) Wheat grain K uptake (kg ha^−1^) = 0.4173 (wheat straw K uptake; kg ha^−1^) + 2.256, R^2^ = 0.9525*, *p* < 0.05

### 3.3 Partial factor productivity and reciprocal internal use efficiency of K by rice

The PFP of rice was significantly lower for the treatments with wheat residue retention (CTR_W25_+GM and CTR_W25_), compared with those with residue removal (CTR_W0_ and CTR_W0_+GM) (Table 2). Green manuring during the intervening period did not significantly change the PFP of rice, regardless of the respective wheat residue retention treatments. Wheat residue retention (CTR_W25_) significantly increased the RIUE_K_ of rice by ∼13.9% than with CTR_W0_. As compared with CTR_W0_, CTR_W0_+GM significantly increased the RIUE_K_ of rice by 3.8 kg Mg^−1^ grain yield (∼29.5%). Green manuring during the intervention period following wheat residue retention (CTR_W25_+GM) increased the RIUE_K_ of rice by 3.3 kg Mg^−1^ grain yield (∼22.4%), compared with no-GM (CTR_W25_). Rice residue removal (CTW_R0_ and/or ZTW_R0_) did not significantly change the RIUE_K_ in rice, but with residue retention (ZTW_R0_), RIUE_K_ in rice was increased by ∼9.2–14.7% (Table 2).

### 3.4 Wheat grain and straw yield, and harvest index

Alone wheat residue retention and/or GM during the intervening period did not significantly change the wheat grain yield (Table 3). However, wheat residue retention and GM during the intervening period (CTR_W25_+GM) significantly increased the wheat grain yield by ∼6.4%, compared to no-GM treatment (CTR_W25_). As compared with CTR_W0_, CTR_W25_+GM increased the wheat grain yield by ∼10.2%. Zero tillage with complete rice residue removal (ZTW_R0_) significantly decreased the wheat grain yield by ∼7.3% than with CTR_W0_. However, rice residue retention (ZTW_R100_) significantly increased the wheat grain yield by ∼16.9%, compared with ZTW_R0_. Wheat straw yield response to different tillage, GM and crop residue management treatments was similar to that for wheat grain yield (Table 3). The CTR_W25_+GM significantly increased the wheat straw yield by ∼6.3% than with CTR_W25_ treatment. The HI for wheat did not differ significantly for the compared treatments.

**Table 3:** Effect of tillage, crop residue management and green manure on grain yield, straw yield, and harvest index (HI), K uptake by grains and straw and K use efficiency indices viz. partial factor productivity (PFP) and reciprocal internal use efficiency (RIUE_K_) for wheat in north-western India.

Treatments^a^	Wheat grain yield (Mg ha^−1^)	Wheat straw yield (Mg ha^−1^)	Harvest index (HI)	Grain K uptake (kg ha^−1^)	Straw K uptake (kg ha^−1^)	Total K uptake (kg ha^−1^)	PFP	RIUE_K_ (kg Mg^−1^ grain yield)
Rice establishment								
CTR_W0_	4.98a ± 0.13^b^	5.53a ± 0.23	0.47a ± 0.01	21.1a ± 1.5	45.7a ± 3.4	67.3a ± 4.8	148a ± 10	13.3a ± 1.0
CTR_W25_	5.16a ± 0.16	5.56a ± 0.27	0.48a ± 0.01	22.7b ± 1.5	49.1b ± 4.4	71.7b ± 5.9	151a ± 13	13.7a ± 0.9
CTR_W0_+GM	5.37 ab ± 0.21	5.86 ab ± 0.28	0.48a ± 0.01	25.0c ± 2.1	53.9c ± 5.4	78.9c ± 7.5	155 ab ± 9	14.4b ± 1.2
CTR_W25_+GM	5.49b ± 0.31	5.91b ± 0.39	0.48a ± 0.01	26.9d ± 3.2	58.5d ± 7.4	85.4d ± 10.6	159b ± 11	15.1c ± 0.9
Wheat establishment								
CTW_R0_	5.23b ± 0.18	5.74b ± 0.26	0.48a ± 0.01	23.4b ± 1.9	51.5b ± 4.7	75.4b ± 6.6	209b ± 14	14.1b ± 1.2
ZTW_R0_	4.85a ± 0.23	5.32a ± 0.32	0.48a ± 0.01	20.5a ± 2.1	44.6a ± 5.2	65.1a ± 7.3	194b ± 15	13.1a ± 0.9
ZTW_R100_	5.67c ± 0.19	6.15c ± 0.28	0.48a ± 0.01	27.7c ± 2.2	59.4c ± 5.5	87.0c ± 7.7	57a ± 5	15.1c ± 1.2

Mean values for different treatments for each crop establishment method followed by different letters are significantly different at *p* < 0.05 by least significant difference (LSD) test.

a Refer Table 1 for treatment details.

b Values indicate standard error from mean.

### 3.5 Grain, straw and total K uptake by wheat

Wheat residue retention (CTR_W25_) significantly increased wheat grain K uptake by ∼7.6%, compared with CTR_W0_ (Table 3). However, GM during the intervening period (CTR_W0_+GM) increased the wheat grain K uptake by ∼18.5%, compared with CTR_W0_. Significant increase in grain K uptake by ∼18.5% in CTR_W25_+GM treatment than with CTR_W25_ was observed. Rice residue removal in ZTW_R0_ treatment has significantly lower wheat grain K uptake than with CTW_R0_, due to noteworthy yield loss (∼7.3%) under ZTW_R0_. Similar to rice, a linear significant relationship between wheat grain and straw K uptake was observed (Fig. 1). The relationship between grain and straw K uptake by wheat could best be described by Eq. (5).

(5) Wheat grain K uptake (kg ha^−1^) = 0.4173 (wheat straw K uptake; kg ha^−1^) + 2.256, R^2^ = 0.9525*; *p* < 0.05

**Fig. 2:**
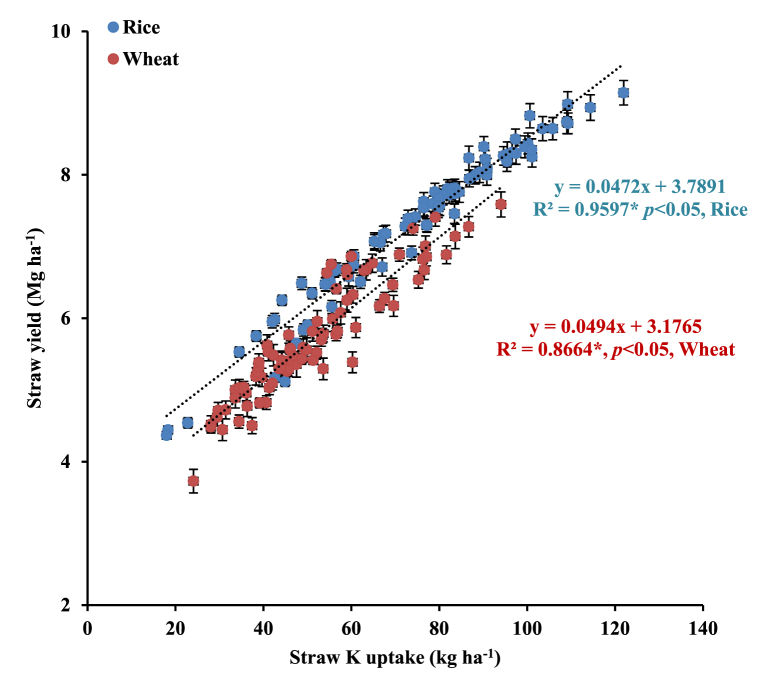
Relationship between straw K uptake and straw yield of rice and wheat in north-western India. *Error bars indicate standard error from mean*. Data pooled for 7-years (2011/12–2017/18). These polynomial relationships are described using different symbols; red solid dots for wheat straw yield vs. straw K uptake by wheat and blue solid dots for rice straw yield vs. straw K uptake by rice.

Fig. 2 illustrates that straw K uptake linearly increased the crops’ (rice/wheat) straw yield. The relationship between straw yields as a function of straw K uptake could be described by straight line function (Eqs. (6) and (7)).

(6) Rice straw yield (Mg ha^−1^) = 0.0472 (rice straw K uptake; kg ha^−1^) + 3.7891, R^2^ = 0.9597*; *p* < 0.05

(7) Wheat straw yield (Mg ha^−1^) = 0.0494 (wheat straw K uptake; kg ha^−1^) + 3.1765, R^2^ = 0.8664*; *p* < 0.05

### 3.6 Partial factor productivity and reciprocal internal use efficiency of K by rice

The PFP of rice was significantly lower for the treatments with wheat residue retention (CTR_W25_+GM and CTR_W25_), compared with those with residue removal (CTR_W0_ and CTR_W0_+GM) (Table 2). Green manuring during the intervening period did not significantly change the PFP of rice, regardless of the respective wheat residue retention treatments. Wheat residue retention (CTR_W25_) significantly increased the RIUE_K_ of rice by ∼13.9% than with CTR_W0_. As compared with CTR_W0_, CTR_W0_+GM significantly increased the RIUE_K_ of rice by 3.8 kg Mg^−1^ grain yield (∼29.5%). Green manuring during the intervention period following wheat residue retention (CTR_W25_+GM) increased the RIUE_K_ of rice by 3.3 kg Mg^−1^ grain yield (∼22.4%), compared with no GM (CTR_W25_). Rice residue removal (CTW_R0_ and/or ZTW_R0_) did not significantly change the RIUE_K_ in rice, but with residue retention (ZTW_R0_), RIUE_K_ in rice was increased by ∼9.2–14.7% (Table 2).

### 3.7 Sustainable yield index for rice and wheat

**Fig. 3:**
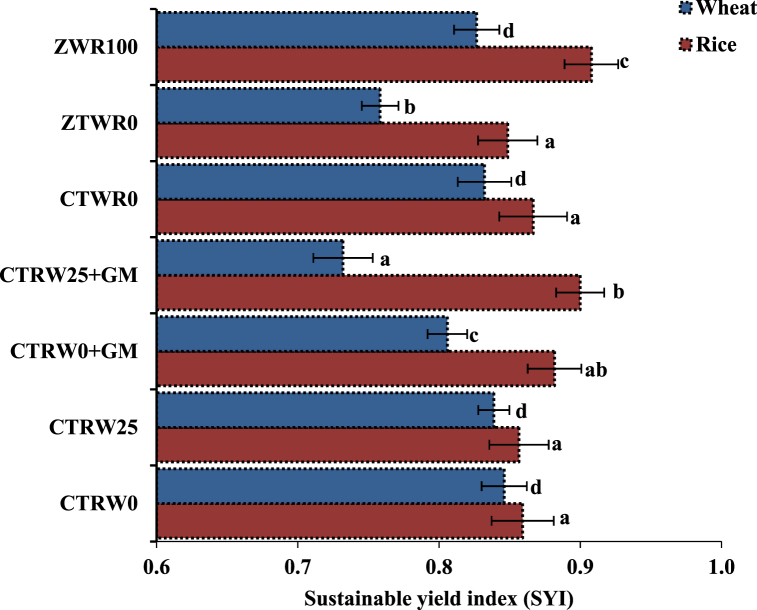
Effect of tillage, crop residue management, and green manure on sustainable yield index (SYI) of rice and wheat in north-western India. *Error bars indicate standard error from mean*. The mean values for different treatments for each crop establishment method followed by different letters are significantly different at *p* < 0.05 by least significant difference (LSD) test.

The SYI for rice varied between 0.85 and 0.91 Mg ha^−1^ and between 0.73 and 0.85 Mg ha^−1^ for wheat (Fig. 3). Wheat residue retention (CTR_W25_) did not significantly influence the SYI for rice, compared with residue removal (CTR_W0_). Green manuring during the intervening period (CTR_W0_+GM) did not significantly increase the SYI for rice, compared with CTR_W25_ and/or CTR_W0_. Zero tillage with residue retention (ZTW_R100_) significantly increased the SYI for rice by ∼7.1% than with residue removal (ZTW_R0_). Unlike rice, the SYI for wheat was significantly decreased by ∼5.8% with GM during intervening period (CTR_W0_+GM) as compared to CTR_W0_. The ZTW_R100_ increased the SYI for wheat by ∼9.2%, compared with ZTW_R0_. The CTW_R0_ increased the SYI for wheat by ∼9.2% than with ZTW_R0_ treatment.

### 3.8 Crop yields and K uptake

**Fig. 4:**
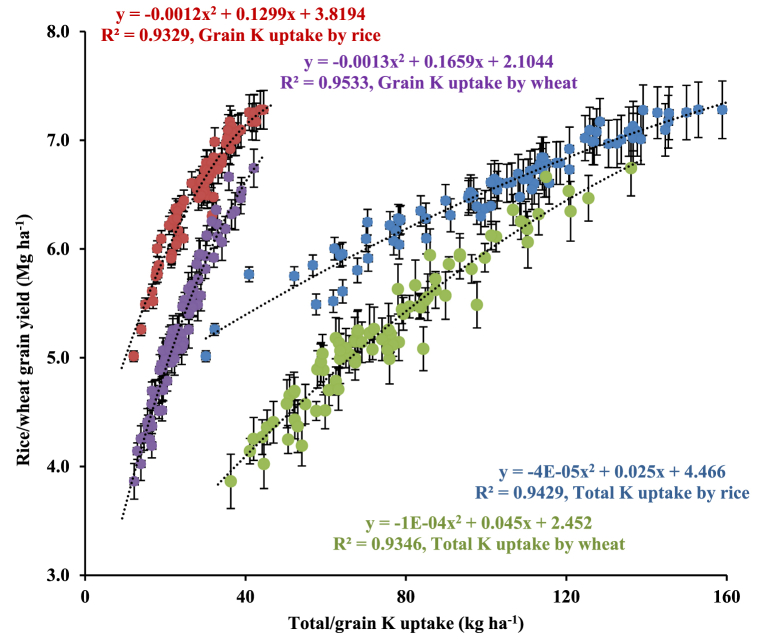
Relationship between total and grain uptake and grain yield of rice and wheat in north-western India. *Error bars indicate standard error from mean*. Data pooled for 7- years (2011/12–2017/18). These polynomial relationships are described using different symbols; red solid dots for rice grain yield vs. grain K uptake by rice, purple solid dots for wheat grain yield vs. grain K uptake by wheat, blue solid dots for rice grain yield vs. total K uptake by rice, and green solid dots for wheat grain yield vs. total K uptake by wheat.

Fig. 4 illustrates the relationship between total K uptake vis-à-vis crops’ (rice and wheat) grain yield. These relationships revealed a polynomial increase in crop grain yields with grain and total K uptake. The relationship between rice grain yield as a function of grain K uptake and total K uptake was described by Eqs. (8) and (9).

(8) Rice grain yield (Mg ha^−1^) = −0.0012(rice grain K uptake; kg ha^−1^)^2^ + 0.1299 (rice grain K uptake; kg ha^−1^) + 3.8194, R^2^ = 0.9329

(9) Rice grain yield (Mg ha^−1^) = -4E-05 (total K uptake; kg ha^−1^)^2^ + 0.025 (total K uptake; kg ha^−1^) + 4.466, R^2^ = 0.9429

Similarly, these relationships for wheat could be described by Eq. (10)-11).

(10) Wheat grain yield (Mg ha^−1^) = −0.0013 (wheat grain K uptake; kg ha^−1^)^2^ + 0.1659 (wheat grain K uptake; kg ha^−1^) + 2.1044, R^2^ = 0.9533

(11) Wheat grain yield (Mg ha^−1^) = -1E-04 (total K uptake; kg ha^−1^)^2^ + 0.045 (total K uptake; kg ha^−1^) + 2.452, R^2^ = 0.9346

### 3.9 Partial K budget for rice-wheat system

The partial K budget for rice-wheat cropping system established under different tillage, crop residue management and green manure treatments was estimated based on total K added and total K uptake in 7- years of continuous cropping (Table 4). The estimated total K addition in 7-years varied between 175 and 361 kg K ha^−1^ for rice and between 175 and 706 kg K ha^−1^ in wheat. In a cropping system, total K added varied between 508 and 979 kg K ha^−1^. The total K uptake (grains + straw) in 7-years varied between 569 ± 24 and 870 ± 52 kg K ha^−1^ for rice and between 467 and 609 kg K ha^−1^ for wheat, totalling between 1036 ± 78 and 1468 ± 91 kg K ha^−1^ for rice-wheat cropping system. These results revealed that apparent K balance for a cropping system was net negative; varied between −809 and −428 kg K ha^−1^. Although, the apparent K balance was net negative for treatments having residue (CTR_W25_, ZTW_R100_), yet showed reduced K mining by 56–262 kg K ha^−1^ (∼11.9–61.2%) for CTR_W25_ and ZTW_R100_ over their respective treatments with residue retention (CTR_W0_ and ZTW_R0_).

**Table 4:** Balance sheet of total fertilizer-K added, total K uptake and K balance under different tillage, crop residue management and green manure treatments in soil after 7-years (2011/12–2017/18) of rice-wheat cropping in north-western India.

Treatments^a^	Total K added to rice (kg ha^−1^)	Total K added to wheat (kg ha^−1^)	Total K added to rice + wheat system (kg ha^−1^)	Total K uptake by rice (kg ha^−1^)	Total K uptake by wheat (kg ha^−1^)	Total K uptake by rice + wheat system (kg ha^−1^)	Apparent K balance (kg ha^−1^)
Rice establishment							
CTR_W0_	175	333	508	569a ± 24^b^	467a ± 32	1036a ± 78	−528c ± 24
CTR_W25_	353	347	700	670b ± 29	502b ± 29	1172b ± 84	−472d ± 31
CTR_W0_+GM	175	362	537	794c ± 43	552c ± 26	1346c ± 79	−809a ± 26
CTR_W25_+GM	361	367	728	870d ± 52	598d ± 30	1468d ± 91	−740b ± 32
Wheat establishment							
CTW_R0_	266	175	411	711b ± 38	524b ± 26	1235b ± 86	−794a ± 40
ZTW_R0_	260	175	435	669a ± 41	456a ± 28	1125a ± 82	−690b ± 38
ZTW_R100_	273	706	979	797c ± 37	609c ± 37	1406c ± 101	−428c ± 34

Mean values for different treatments for each crop establishment method followed by different letters are significantly different at p < 0.05 by Least Significant Difference (LSD) test.

a Refer Table 1 for treatment details.

b Values indicate standard error from mean.

### 3.10 Total K and fractions in soil after 7-years of rice-wheat cropping

**Fig. 5:**
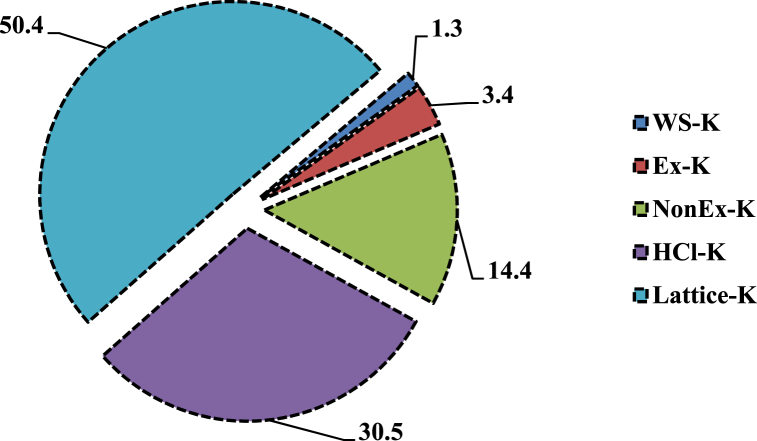
Relative preponderance of different K fractions viz. water soluble-K (WS–K), exchangeable-K (Ex-K), non-exchangeable K (Non Ex-K), hydrochloric acid extractable K (HCl–K) and lattice-K in soils after 7-years (2011/12–2017/18) of rice-wheat cropping in north-western India. *Data pooled for different treatments.*

**Fig. 6:**
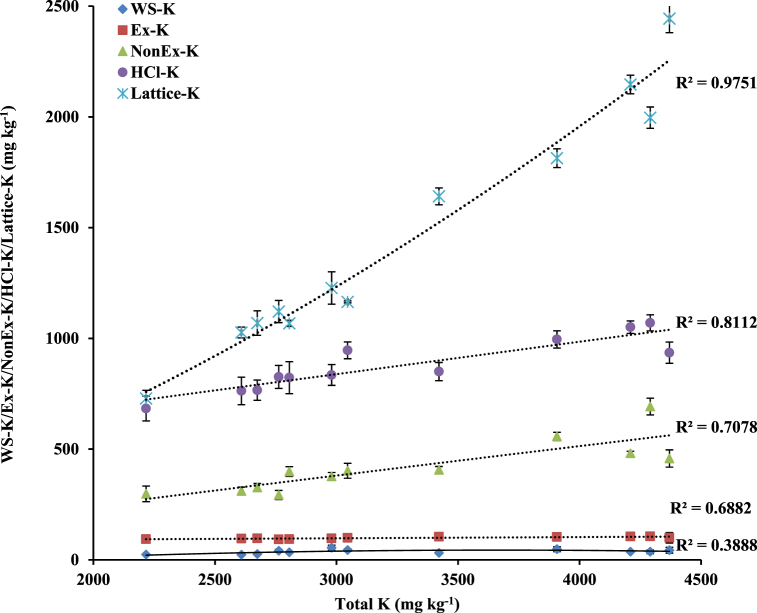
Relationship between total K and water soluble-K (WS–K), exchangeable-K (Ex-K), non exchangeable K (Non Ex-K), hydrochloride extractable-K (HCl–K) and lattice-K in soils after 7-years (2011/12–2017/18) of rice-wheat cropping in north-western India. *Error bars indicate standard error from mean*. Data pooled for different treatments.

**Fig. 7:**
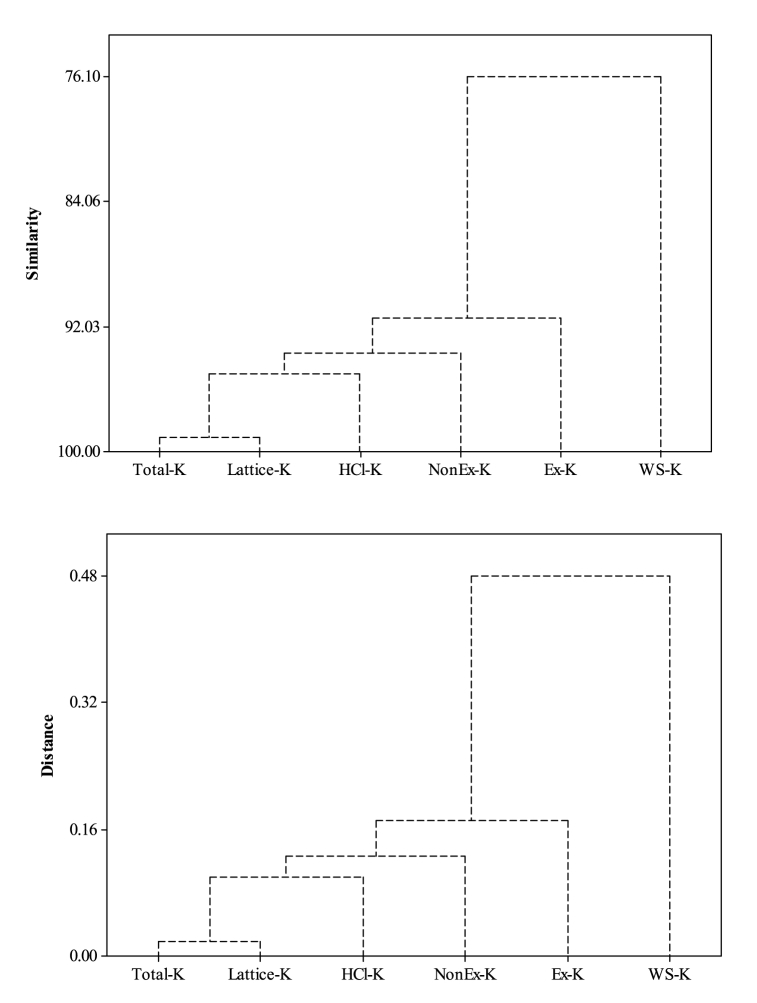
Similarity and single linkage correlation distance between different K fractions of in soils after 7-years (2011/12–2017/18) of rice-wheat cropping in north-western India. *Data pooled for different treatments.*

Total K pool was significantly enlarged by 871 mg kg^−1^ (∼41.3%) with wheat residue retention (CTR_W25_) for 7-years, compared with CTR_W0_ (Table 5). Green manuring during the intervening period in wheat residue retained treatment (CTR_W25_+GM) increased the total K pool by 1248 mg kg^−1^ (∼59.1%) than with CTR_W0_. Zero tillage with rice residue retention (ZTW_R100_) resulted in significant enrichment of total K pool by 656 mg kg^−1^ (∼23.1%), compared with ZTW_R0_. The ZTW_R0_ treatment increased total K pool by 522 mg kg^−1^ (∼22.5%) than the CTW_R0_. The relative preponderance of different K fractions revealed the dominance of lattice-K (∼50.4% of total K pool), followed by HCl–K (∼30.5%) and Non Ex-K (∼14.4%) (Fig. 5). Water soluble K comprised the smallest fraction (∼1.3% of total K), and was significantly higher in treatments with wheat residue incorporation (CTR_W25_/CTR_W25_+GM) as compared to CTR_W0_. Green manuring (CTR_W0_+GM) significantly increased the WS-K fraction by ∼39.5% than with CTR_W0_. Ex-K (∼3.4% of total K) did not differ significantly among treatments. All other fractions viz. Non-Ex-K, HCl–K and lattice-K were significantly improved with wheat residue incorporation and GM. The ZTW_R100_ significantly increased these fractions by ∼18.6, 11.0 and 34.1%, respectively compared to ZTW_R0_. These fractions of differential solubility exhibited an increase with increased total K pool (R^2^ = 0.659–0.975) in soil after 7-years of rice-wheat cropping under different tillage, crop residue management and green manure treatments (Fig. 6). Dendrogram exhibited high correlation single linkage among different K fractions. Total K showed highly significant closely relationship with lattice-K (r = 0.982**, *p* < 0.01), HCl–K (r = 0.901**, *p* < 0.01) and Non Ex-K (r = 0.841**, *p* < 0.01) (Fig. 7). The Non-Ex-K and Ex-K fractions were closely linked and eventually influence WS-K fraction in soil.

**Table 5:** Effect of tillage, crop residue management and green manure on total K and different K fractions viz. water soluble-K (WS–K), exchangeable-K (Ex-K), non-exchangeable K (NonEx-K), hydrochloride extractable K (HCl–K) and lattice-K in soils after 7-years (2011/12–2017/18) of rice-wheat cropping in north-western India.

Treatments^a^	Total K (mg kg^−1^)	Water soluble K (WS–K) (mg kg^−1^)	Exchangeable-K (Ex-K) (mg kg^−1^)	Non exchangeable-K (Non Ex-K) (mg kg^−1^)	Hydrochloride extractable K (HCl–K) (mg kg^−1^)	Lattice-K (mg kg^−1^)
Rice establishment						
CTR_W0_	2110a ± 97^b^	24.3a ± 1.8	95.4a ± 4.8	311.6a ± 27.4	737.3a ± 56	914a ± 84
CTR_W25_	2981b ± 58	46.0c ± 1.6	96.3a ± 8.8	375.8b ± 26.9	865.3b ± 63	1598b ± 71
CTR_W0_+GM	3089b ± 99	33.9b ± 2.1	100.6a ± 9.4	428.4c ± 31.8	907.8c ± 69	1618c ± 87
CTR_W25_+GM	3358c ± 57	42.8c ± 3.2	102.8a ± 9.9	550.4d ± 48.9	1003.8d ± 71	1658d ± 88
Wheat establishment						
CTW_R0_	2318a ± 75	35.5a ± 2.9	94.5a ± 7.9	347.9a ± 34.6	819.5a ± 81	1021a ± 75
ZTW_R0_	2840b ± 98	39.2b ± 3.0	100.1a ± 10.3	412.5b ± 49.6	860.7b ± 74	1427b ± 81
ZTW_R100_	3496c ± 101	35.6a ± 2.4	101.9a ± 9.9	489.3c ± 43.4	955.5c ± 86	1914c ± 98

Mean values for different treatments for each crop establishment method followed by different letters are significantly different at p < 0.05 by Least Significant Difference (LSD) test.

a Refer Table 1 for treatment details.

b Values indicate standard error from mean.

### 3.11 Correlation matrix illustrating relationships K fractions, uptake and crop yields

The Ex-K significantly influence total K uptake by rice (r = 0.733**, *p* < 0.01), rice straw K uptake (r = 0.747**, *p* < 0.01), rice grain K uptake (r = 0.587*, *p* < 0.05), which eventually significantly contributes towards rice grain yield (r = 0.691*, *p* < 0.05) (Table 6). The non-significant (*p* < 0.05) relationship between WS-K fraction and crop K uptake was noteworthy. Rice straw and grain K uptake were significantly related (r = 0.979**, *p* < 0.01). Similarly, wheat straw and grain K uptake were significantly correlated (r = 0.993**, *p* < 0.01). Total K uptake by rice and wheat significantly influence crop grain yields.

**Table 6:** Correlation matrix depicting relationship between K fractions, K uptake and crop yields under different tillage, crop residue management and green manure treatments. (Acronyms: WS-K = water soluble K, Ex-K = exchangeable K).

Variables	WS-K	Ex-K	Total K uptake by rice	Total K uptake by wheat	Rice straw K uptake	Wheat straw K uptake	Rice grain K uptake	Wheat grain K uptake
Total K uptake by rice	0.372	0.733**						
Total K uptake by wheat	0.138	0.569	0.876**					
Rice straw K uptake	0.377	0.747**	0.999**	0.872**				
Wheat straw K uptake	0.157	0.571	0.887**	0.999**	0.883**			
Rice grain K uptake	0.353	0.680*	0.988**	0.871**	0.979**	0.881**		
Wheat grain K uptake	0.150	0.587*	0.873**	0.995**	0.869**	0.993**	0.869**	
Rice grain yield	0.272	0.691*	0.981**	0.895**	0.976**	0.904**	0.979**	0.893**

**Correlations significant at *p* < 0.01, *Correlations significant at *p* < 0.05.

## 4 Discussion

### 4.1 Effect of tillage, green manure and residue management practices on crop yield

In the present study, rice and wheat yield increased by ∼5.3–6.7% and 10.2–16.9%, respectively under CTR_W25_+GM/ZTW_R100_ than CTR_W0_-GM/ZTW_R0_ consistent indicating that the benefits of crop residue retention and GM in terms of crop yield and soil fertility indices. These results confirmed that adoption for major cereal-legume based cropping systems increased crop productivity, farm profitability, moisture retention characteristics, nutrient use efficiencies, and soil fertility [[50], [51], [52], [53]]. A recent global meta-analysis concluded that legumes performed better under no-till system, while among the cereals, wheat yield was less negatively impacted by ZT practices relative to the rice yields [54]. The increased crop productivity has been related to the improvement in hydrothermal regimes, root growth and proliferation, increased soil C and nutrients, and soil improvement in soil structure [5,55,56]. A long-term (7-years) field experiment on a clay loam soil showed that ZT with residue retention (ZTW + R) increasing system productivity by ∼0.63 Mg ha^−1^ year^−1^ than CTW-R [18]. The increased crop productivity was ascribed to better seed germination and rooting due to improved soil physical properties [57,58]. Arora et al. [59] reported lower yield of ZTW-R than CTW-R on a sandy loam soil which was due to poor root growth and decreased to access of wheat roots to soil N. The possible causes for higher wheat grain yield under ZTW + R include the effects of the straw mulch on enhancing the duration of crops’ vegetative growth stage as a result of decreased soil temperature, reduction of soil evaporation increasing soil moisture content, and reduced canopy temperature particularly at grain filling stage due to increased soil moisture conservation [60,61]. The impacts of straw mulch are observed early, even starting from the first crop, whilst significant improvements to soil fertility are discernible in long period of time [15,62,63]. These results suggest that it may take about 4–5 years to observe appreciable yield gain benefits of ZT + R in rice [9,15],. Higher wheat yield under ZT with residue retention compared with the conventional tillage in eastern IGPs has been reported [64,65]. Gathala et al. [66] observed 9–10% higher yield under ZTW + R than ZTW-R than CTW-R.

### 4.2 Effect of tillage, GM and residue management practices on K fractions

Decomposition of rice straw return to soils increases the soil K supply and maintaining high apparent soil K balance [67]. The K^+^ ions released during the process of decomposition binds to the exchange sites having high affinity for K^+^; which is desirable for crops as K^+^ ions are stored in soil and remain protected from leaching [68,69]. Nonetheless, the slower decomposition of crop residue retained as mulch [70,71] prevent rapid K leaching and thereby increases K availability for better root proliferation [63,72]. Increased root growth of cereal crops under un-puddled e.g. ZT condition has been ascribed to increased number of macro-pores and improved aggregation which facilitate root growth, and thereby enable crop to absorb available K from the deeper soil layers [73,74]. Field experiments showed the positive influence of *in-situ* straw management in maintaining high K-supply by influencing available K fractions in soils, than CT without residues [19,74]. Crop residues as mulch act as an insulating layer and reflect incoming solar radiation to reduce the heat flux into and out of the soil as compared to the CT without residues [74]. Nonetheless, higher soil moisture generally observed in the surface layers under residue retention treatment compared with the CT without residues increases the thermal capacity of soil and reduces soil temperature [75,76]. Consistent to the results of previously published studies [19,77], we also observed higher K availability in ZT as compared with CT. Relatively higher Ex-K content under residue retention treatments compared with CT (without residue) is explainable in view of (i) residue inversion leading to greater soil-residue contact during tillage, (ii) a microbial spurt releasing C and other plant nutrients resulting in faster decomposition of residue, and (iii) decreased K leaching through mass flow [78]. The organic acids released during crop residues decomposition dissociate to produce organic anions with net negative electric charges developed in soils. These organic compounds prefer adsorbing cations with a higher positive charge (Al^3+^, Ca^2+^ and Mg^2+^), leaving K^+^ to be adsorbed by the negatively charged colloids. It helps reduce leaching losses of K^+^ ions in soil profile under ZT [79].

The reduced tillage intensity and residue retention as mulch increased available K in surface soil [15,80]. Straw contains a large amount of K, therefore *in-situ* straw management would be the most promising measure to deal with soil K deficiency [12,81]. Jat et al. [14] reported higher available N, P and K under CA in northwest India. As K depletion can promote the conversion of Non-Ex-K to WS-K and Ex-K, more negative K balances were accompanied with K deficiency soils [82,83]. The plant available K forms that contribute largely towards crops’ K uptake remain in a dynamic equilibrium with Non-Ex-K pool [84]. The available forms of K determine the equilibrium between the soil solution K and the K associated with ion exchange and interlayer surfaces [85]. Non-exchangeable pool serves as K reserves that slowly become available overtime, and significantly influence K availability in soils [86]. The decline in all forms of K under CT-R was due to the excess removal of K by crops from external sources. The increase in the exchangeable K under ZT + R could be due to an increase in the exchange sites and Non-Ex-K in clay interlayers can be trapped by plant roots, especially in monocots [[87], [88]]. Wihardjaka et al. [84] and Poss et al. [89] observed a positive balance of K on *in-situ* management of crop residue and suggested that adequate supply of K into the soil can be maintain either return the crop residue back or apply more K externally.

## 5 Conclusion

Medium-term field experiment studies suggested that adoption of conservation agricultural practices (cereal-green manure rotation) improved a crop yield, potassium use efficiencies, potassium fractions and potassium balance compared to conventional tillage. Our results demonstrate that tillage, green manuring and crop residue management is feasible in sandy loam soils of India without any yield penalty for the first 7-years and subsequently with 6.7–16.9% yield increase. Recycling of K-rich cereal residues is an effective way to balance soil K depletion for sustaining the crops yield under intensive rice–based systems in the region. The outcomes of this experimental delivers an evidence for academician, researcher, extension specialists, policy leaders, and farmers to drive its wider adoption of cereal-green manure rotation to address the environmental quality and sustainable development issues under RWS in north-western India.

## Author contribution statement

Sandeep Sharma; Pritpal Singh: Conceived and designed the experiments; Performed the experiments; Analysed and interpreted the data; Contributed reagents, materials, analysis tools or data; Wrote the paper.

Manzer H Siddiqui; Saud Alamri; Javed Iqbal: Analysed and interpreted the data; Wrote the paper.

## Data availability statement

Data included in article/supp. material/referenced in article.

## Declaration of competing interest

The authors declare that they have no known competing financial interests or personal relationships that could have appeared to influence the work reported in this paper.
